# Microstructure Evolution, Hardness, and Tribological Behaviors of Ti-50.8Ni SMA Alloy with Ultrasonic Surface Shot Peening Treatment

**DOI:** 10.3390/ma17112644

**Published:** 2024-05-30

**Authors:** Zihan Chen, Xuanpeng Li, Yong Li, Yu Wang, Yongxin Jian

**Affiliations:** 1 State Key Laboratory of Oil and Gas Equipment, CNPC Tubular Goods Research Institute, Xi’an 710077, China; chenzihan@cnpc.com.cn (Z.C.);; 2 Xi’an Thermal Power Research Institute Co., Ltd., Xi’an 710032, China; 3 Shaanxi Special Equipment Inspection and Testing Institute, Xi’an 710048, China; 4 School of Mechanical Engineering, Xi’an Jiaotong University, Xi’an 710049, China

**Keywords:** TiNi SMA, USSP, microstructure, hardness, tribological behavior

## Abstract

To explore a new method to improve the wear resistance of TiNi shape memory alloy (SMA), Ti-50.8Ni alloy was treated by the method of ultrasonic surface shot peening. The microstructure evolution, hardness, and tribological behaviors have been further investigated to evaluate the effect of ultrasonic surface shot peening (USSP). The surface microstructure can be refined to some extent while the basic phase composition has little change. USSP can facilitate the martensitic transformation in the surface layer, which benefits improving the surface hardness. Additionally, the hardness of Ti-50.8Ni alloy increases first and then decreases with the increase of applied load, but the USSP-treated alloy tends to be more sensitive to load. USSP treatment can improve the wear resistance and reduce the coefficient of friction (COF) in case of a low sliding wear speed of 5 mm/s. However, the tribological properties of USSP-treated alloy are reversely worse in the case of 10 mm/s. This is mainly attributed to the combined effect of stress-induced martensite transformation and degeneration resulting from the frictional heating during the dry sliding wear process.

## 1. Introduction

TiNi alloys are well known as the typical shape memory alloys (SMAs), possessing distinctive superelasticity and shape memory effect [1,2,3]. In addition, TiNi alloys have excellent mechanical properties and good biocompatibility. Thus, this kind of alloy shows extensive applications in the fields of aerospace, automotive, and biomedicine [4]. Compared with other conventional alloys, TiNi SMA shows superior wear-resistant performance, which is mainly attributed to the pseudo-elasticity [5,6,7]. In addition, the good work hardening and fatigue resistance also make great contributions to its wear resistance [8]. In spite of the considerable wear resistance, the Ni-rich debris generated during the wear process still limits the application of the TiNi alloy [9]. Therefore, the hot-point issues have always been to improve the friction and wear properties of TiNi SMA [10].

Until now, plenty of studies have been conducted to explore the wear behaviors of TiNi SMA. Farhat et al. [11] evaluated the sliding wear properties of TiNi SMA, and found the ratio of E/H and elastic recovery ratio determined the superior wear resistance. Wang et al. [12] investigated the microstructure responses and deformation mechanisms of Ti-51.5 Ni alloy during the reciprocating sliding wear process. They found that the martensite phase is the major phase in the wear surface, which was supposed to be beneficial to improve the wear resistance. Meddah et al. [13] found that TiNi exhibited superior wear resistance to TC4 alloy, and Ni content played an important role in the tribological behaviors. Furthermore, Shen et al. [4] evaluated the wear performance of SLM-fabricated Ti47Ni53 alloy and confirmed that the Ni4Ti3 precipitates could improve the alloy’s hardness and wear resistance. Waugh et al. [9] attempted to enhance the wear resistance of the NiTi surface by laser-selective area nitriding. According to their results, the partially nitrided sample with 76% TiN coverage was the alternative suggestion for the surface hardening and wear resistance enhancement compared with the fully nitrided method.

Ultrasonic shot peening (USSP) is an effective surface modification treatment to enhance the wear and corrosion resistance of alloys by introducing severe plastic deformation [14,15]. Han et al. [14] effectively eliminated the localized corrosion of Aluminum alloy by preparing the surface equiaxed nanograins by the method of USSP. Kumar et al. [15] modified the hot corrosion resistance of Ti-6Al-4V by producing the surface nanostructure with the method of USSP. Except for the corrosion behaviors, the surface nanostructure can play an important role in affecting the tribological properties of materials. Zhang et al. [16] investigated the tribological behaviors of AZ31 magnesium alloy after USSP treatment, and confirmed that the treated alloy exhibited better wear resistance and lower coefficient of friction. Likewise, the surface microstructure, as well as the hardness, can also be modified by the USSP method. In this case, it is expected to further improve the tribological performance of TiNi SMA by USSP treatment. However, there have been no public reports investigating the effect of USSP on the tribological behaviors of TiNi alloy.

Therefore, the near-equalatomic Ti-50.8Ni alloy has been selected and treated with USSP to modify the surface to evaluate the effect of the USSP treatment [17]. Furthermore, dry sliding wear behaviors have been investigated systematically to clarify the effect of USSP treatment. The wear mechanisms have also been discussed associating with the wear surface features. According to the obtained results, the USSP treatment is expected to be used to modify the tribological performance of TiNi SMA parts. Additionally, the theory of tribological behaviors of TiNi SMA can also be further enriched.

## 2. Materials and Experimental Methods

### 2.1. Materials

In this work, Ti-50.8Ni alloy was prepared in a vacuum induction furnace with medium frequency. Subsequently, the as-cast alloy was processed through the working procedures of homogenizing annealing-forging-hot rolling-cold rolling-vacuum annealing-cold rolling-vacuum annealing to obtain the TiNi alloy plate with a thickness of 2 mm. The detailed processing methods and parameters have been given in the previous works [18,19].

**Figure 1:**
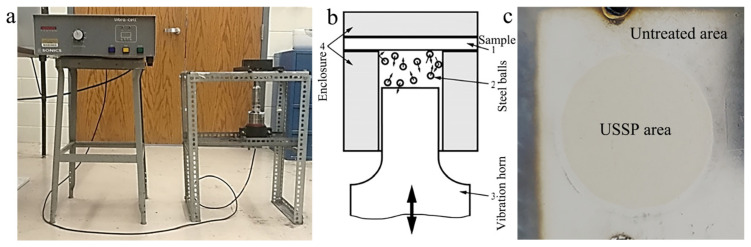
The ultrasonic shot peening treatment setup and the USSP-treated sample: (**a**) Photo of the USSP treatment setup; (**b**) Schematic diagram of the setup; (**c**) Photo of the USSP-treated sample.

The ultrasonic shot peening treatment (USSP) was conducted on a self-designed setup as shown in Figure 1 [16]. The pre-treated TiNi plate was fixed on a cylindrical enclosure with an inner diameter of 25 mm. Then, the stainless steel balls with a diameter of 3 mm were used to impact the sample surface under the interaction of an ultrasonic vibration horn. The vibration frequency during the USSP treatment was 20 kHz, and the distance from the top surface of the horn to the sample surface was 10 mm. The duration for the USSP treatment is 3 min for each sample. Figure 1c exhibits the surface of Ti-50.8Ni alloy after USSP treatment, on which the USSP-treated surface is much brighter.

**Figure 2:**
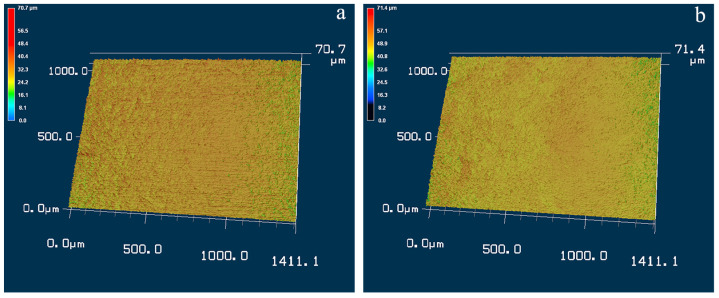
3D morphologies of the surfaces of Ti-50.8Ni samples: (**a**) without USSP treatment; (**b**) after USSP treatment.

Figure 2 shows the three-dimensional surface micrographs of Ti-50.8Ni alloy before and after the USSP treatment. By comparison, the average roughness of the surface declines slightly after the USSP treatment from 2.253 to 1.974 μm. From Figure 2a, the micro-grooves can be observed on the surface along the rolling direction, which is caused by the processing of the TiNi plate. After the USSP treatment, the sample surface is covered by dense dimples without directional features. During the USSP process, the steel balls repeatably impacted the sample surface to cause the featured surface morphology.

### 2.2. Microstructure and Hardness Testing

The microstructure of Ti-50.8Ni alloy was observed by utilizing a scanning electron microscope (SEM, Hitachi SU3500, Hitachi, Tokyo, Japan). Before the observation, the polished surfaces of two samples were etched by the mixed solution of HF, HNO_3_, and H_2_O with a ratio of 1:3:10 [8]. The phase composition was determined by using X-ray diffraction (XRD) on a BRUKER D8 Advance diffractometer (Bruker, Billerica, MA, USA) with the Cu-Kα radiation. The voltage and electricity were set as 40 kV and 30 mA, respectively. The specimen was scanned in the 2θ ranging from 10° to 90° with a step-scan mode (0.02° per step). In addition, electron backscatter diffraction (EBSD, Oxford, UK) was used to help confirm the phase evolution and crystal grain features. On the other hand, the hardness testing was conducted on an HVS-50ZYD Vickers hardness tester (Lihua, Jinan, China) with a test load ranging from 10 to 100 g. For each sample, fifteen measurements were performed in arbitrary areas to acquire the average value.

### 2.3. Dry Sliding Wear Testing

The wear resistance of the Ti-50.8Ni alloy before and after USSP treatment was tested by a ball-on-disk dry sliding wear testing machine. GCr 15 ball was selected as the counter ball, which holds a hardness of 700 HV. During the wear process, the ball slid reciprocatively on the surface of the tested alloy with a stroke length of 10 mm. Different applied loads (2 and 4 N) and sliding speeds (5 and 10 mm/s) were set to evaluate the tribological performance with wear conditions. The total wear distance for each test was 10 m. The specific testing conditions of dry sliding wear are listed in Table 1. After wear, the volume of the wear track was measured by a color 3D laser scanning microscope (VK-970, Keyence Corporation, Osaka, Japan). For each test condition, at least three samples were tested to guarantee the data replicability. On the other hand, the wear surface morphology was observed by the SEM to help clarify the wear mechanism.

## 3. Results and Discussion

### 3.1. Microstructure Analysis

**Figure 3:**
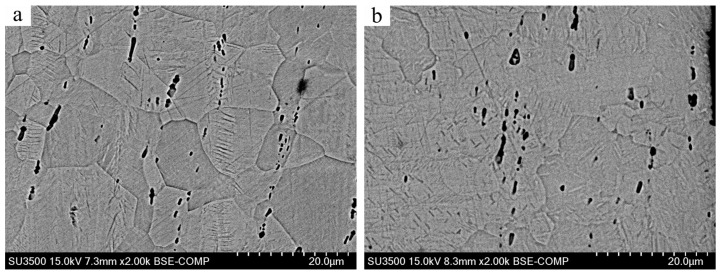
SEM micrographs of Ti-50.8Ni samples: (**a**) As-received; (**b**) After USSP treatment.

Figure 3 shows the microstructure of Ti-50.8Ni alloys before and after the USSP treatment. Firstly, a few micro-voids can be observed inside the alloy samples, which are supposed to be caused during the deformation process. In Figure 3a, these micro-voids seem to distribute with band-like patterns along the rolling direction. However, the distribution of micro-voids tends to be more disordered after USSP treatment. This indicates that the inner defects underlying the surface can be affected by the impact of the steel balls. It is hypothesized that the randomly distributed micro-voids have less influence on the mechanical properties of the alloy. On the other hand, the grains seem to be refined after USSP treatment though the boundaries become fuzzy. During the USSP treatment, the steel balls continually impact the surface of Ti-50.8Ni alloy, which accounts for the evolutions of grain size and morphology [15,20]. Consequently, the surface of the Ti-50.8Ni alloy is supposed to be strengthened with the treatment of USSP.

**Figure 4:**
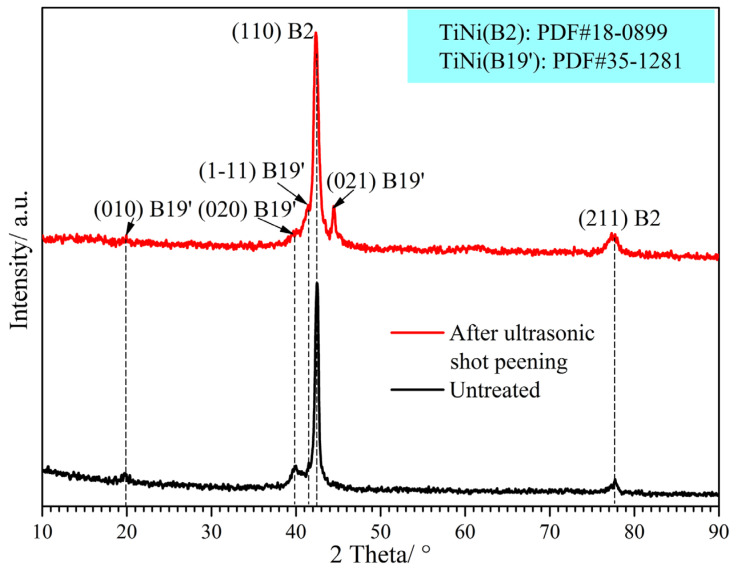
XRD patterns of Ti-50.8Ni samples before and after USSP treatment.

To investigate the effect of USSP on the phase composition, Figure 4 displays the XRD patterns of Ti-50.8Ni alloys before and after the USSP treatment. As shown, the untreated Ti-50.8Ni alloy is mainly composed of B2 parent phase [21,22,23]. In addition, tiny peaks of the B19′ phase can also be detected, which is supposed to be caused by the local segregation [18,24]. However, the diffraction peaks of the B19′ phase are obviously more intensive for Ti-50.8Ni alloy after the USSP treatment even though the B2 phase still dominates [25]. According to the previous result [18], the transformation temperatures from B2 to R and R to B19′ are much lower than the room temperature for Ti-50.8Ni alloy. Thus, the abnormal phase transformation from B2 to B19′ is supposed to be induced by the USSP treatment. In the case of the continual impact of the steel balls, the introduced stress would induce martensite (SIM) transformation. The transformed martensite (B19′) can be retained after USSP due to the retained stresses in the surface layer. The phase transformation originating from the surface deformation is beneficial to improve the surface hardness of Ti-50.8Ni alloy [3]. On the other hand, it can be found that the (110) peak of B2 becomes wider after USSP treatment. According to Scherrer’s equation [26], the broadened diffraction peak generally implies a smaller grain size. Thus, it can be concluded that the grain of the B2 phase can be refined to some degree after USSP treatment. This result agrees well with the microstructure observation in Figure 3.

**Figure 5:**
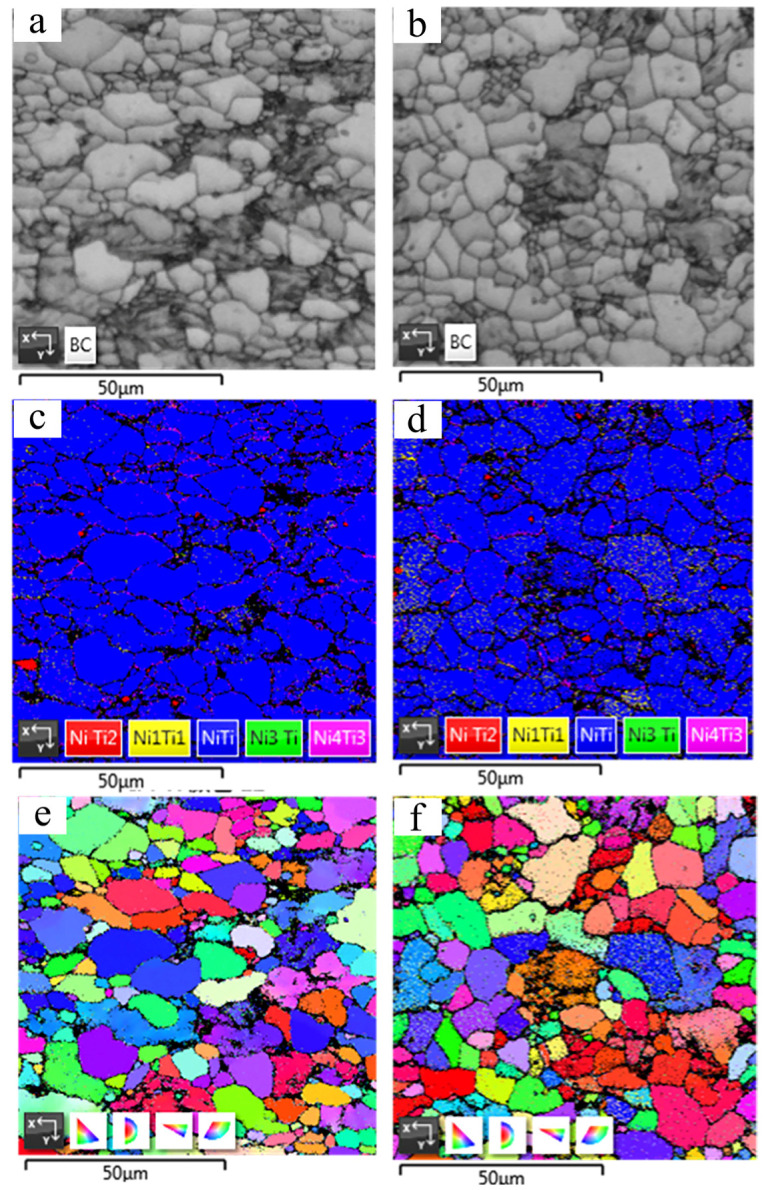
EBSD analysis of Ti-50.8Ni samples before and after the USSP treatment: (**a**) Phase distribution maps of the untreated alloy; (**b**) Phase distribution maps of the USSP-treated alloy; (**c**) Phase distribution maps of the untreated alloy; (**d**) Phase distribution maps of the USSP-treated alloy; (**e**) IPF of the untreated alloy; (**f**) IPF of the USSP-treated alloy.

EBSD analysis has been conducted to further characterize the microstructure evolution of Ti-50.8Ni alloy after USSP treatment. Figure 5c,d are the phase distribution maps of Ti-50.8Ni alloys. Overall, both Ti-50.8Ni alloys are mainly composed of the B2 parent phase, which is consistent with the XRD patterns. In addition, a few Ni4Ti3 precipitates appear along the grain boundaries of the B2 phase. This phenomenon has also been found before in the previous report about the near equiatomic TiNi alloy [19,24]. Additionally, B19′ phase particles can be found to randomly distribute inside the B2 grains for USSP-treated alloy (see the yellow areas). This further confirms the existence of a martensite phase in the surface layer of the USSP-treated Ti-50.8Ni alloy. From Table 2, the content of the B19′ phase is 0.69 and 3.75% in Ti-50.8Ni alloy before and after the USSP treatment, respectively, while other phases have little change. Figure 5e,f display the inverse pole figure (IPF) maps of the B2 phase in the untreated and USSP-treated Ti-50.8Ni alloy. As shown, there is no preferential lattice orientation for the B2 phase in both samples. For the untreated alloy, the possible preferential orientation features of crystals can be eliminated by the annealing process after rolling. The USSP treatment would not cause the apparent evolution of crystal orientation in Ti-50.8Ni alloy. In addition, the grain refinement seems unapparent in the USSP-treated Ti-50.8Ni alloy from the perspective of EBSD images.

### 3.2. Surface Hardness

**Figure 6:**
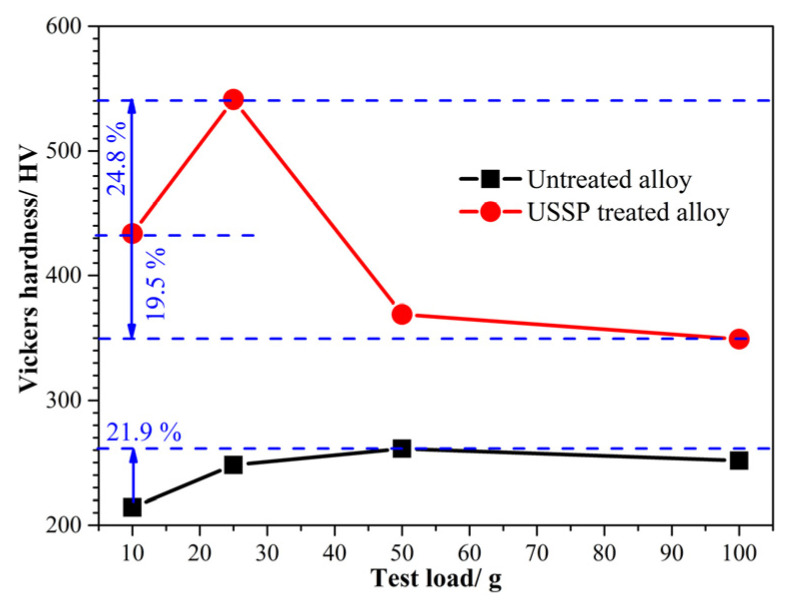
Surface hardness of Ti-50.8Ni alloys before and after USSP treatment.

Hardness is an important factor in influencing the friction and wear properties of materials [16,27,28]. The surface hardness of Ti-50.8Ni alloy has been measured under different loads (Figure 6). For the untreated alloy, the Vickers hardness first increases with the increase of testing load and starts to decline when the load exceeds 50 g. Compared with the hardness tested under 10 g, the highest hardness obtained on the condition of 50 g has been improved by about 21.9%. Considering the superelastic characters of the TiNi alloy, the stress-induced martensite transformation may occur during the indentation process of hardness testing, which is beneficial for improving the hardness [29]. Thus, the higher testing load can induce a higher degree of phase transformation, accounting for the higher hardness [30]. However, excessive indentation deformation would bring about damage to the microstructure, which is reversely detrimental to the alloy’s hardness [31].

For the USSP-treated alloy, the average surface hardness is higher than that of untreated alloy, which is supposed to be mainly attributed to the existence of martensite phase [29,32,33]. In addition, the hardness of the USSP-treated Ti-50.8Ni alloy increases first and then decreases, with the highest hardness obtained under a test load of 25 g. The highest hardness reaches about 540 HV, which is more than two times that of the untreated alloy. After that, the hardness decreases dramatically with the increase of the test load. Compared to that under 10 g, the surface hardness of Ti-50.8Ni alloy decreases by about 19.5% under the test load of 100 g. However, the average hardness is still higher than that of untreated Ti-50.8Ni alloy, indicating the potential to show better wear resistance. After the USSP treatment, the retained martensite phase underlies the surface, contributing to the higher surface hardness of the Ti-50.8Ni alloy. Furthermore, the retained stresses after USSP treatment can facilitate the martensite transformation during indentation so that the hardness improvement is more apparent and quicker. Nevertheless, the tolerance to elastic deformation becomes worse after the USSP treatment, causing the premature hardness to decline. Overall, the hardness variation caused by the applied load is deemed to greatly affect the wear performance [30,34].

### 3.3. Dry Sliding Wear Properties

**Figure 7:**
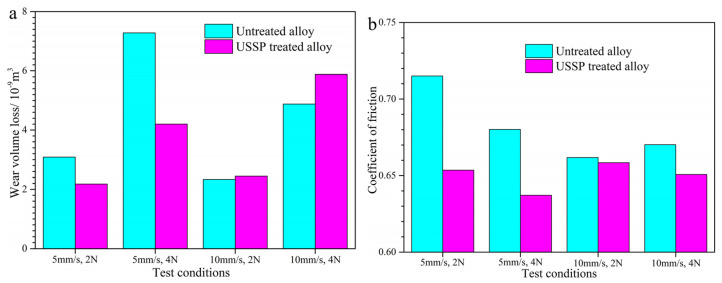
Dry sliding wear properties of Ti-50.8Ni alloys before and after USSP treatment: (**a**) Wear volume loss; (**b**) Coefficient of friction.

Figure 7 shows the wear volume losses and coefficients of friction (COFs) of Ti-50.8Ni alloy before and after the USSP treatment. The wear volume loss and COF show varying trends when the friction condition changes. As shown in Figure 7a, the wear volume loss can be increased with the increase of applied load regardless of the sliding speed. This is reasonable that the removal of materials has a tight relationship with the applied load during the wear process [35]. On the condition of 5 mm/s, the COFs tend to decline with the applied load increased from 2 to 4 N despite the increasing wear volume loss. Generally, a higher applied load would contribute to a higher contact area between the GCr15 ball and the sample surface, which is an important factor in decreasing the COF [36]. This result agrees well with the report of Meddah’s report on the dry sliding wear TiNi alloy [13]. Compared with the untreated alloy, the USSP-treated Ti-50.8Ni alloy shows much lower wear volume loss as well as lower COFs. Considering the lower COF and higher hardness, it is reasonable for the better wear resistance of the USSP-treated alloy [34,37,38]. Furthermore, it can be found that the increment of wear volume loss resulting from the increase of applied load is relatively lower for the USSP-treated alloy. This indicates that the USSP-treated alloy can show a more predominant advantage under the higher applied load on the condition of 5 mm/s. From the result of the hardness analysis, it can be concluded that the hardness improvement is much larger for the USSP-treated alloy with the increase of applied load. This is exactly consistent with the result of wear volume loss. Overall, the USSP-treated alloy shows superior tribological performance on the condition of 5 mm/s.

However, the wear volume loss of the USSP-treated Ti-50.8Ni alloy seems to be a little higher than the untreated alloy on the condition of 10 mm/s. Additionally, the increment of wear volume loss resulting from the increased applied load is larger for the USSP-treated alloy. Based on the characters of pseudoelasticity, the martensite transformation induced by the friction stress would be more apparent under the higher sliding speed of 10 mm/s [1,11,39,40]. Thus, the wear volume loss can be effectively decreased for the untreated alloy on the condition of 5 mm/s. This result agrees well with Farhat’s report that the increasing friction frequency can reduce the wear rate of TiNi alloy due to the hardening effect of martensite transformation [11]. However, the wear volume loss tends to increase when the sliding speed increases from 5 to 10 mm/s. This indicates that the USSP-treated alloy shows lower wear resistance under higher sliding speed. Even though the surface hardness of the Ti-50.8Ni alloy can be improved by the USSP treatment, the superelastic effect on the wear resistance seems to be weakened under higher sliding speed. In reverse, the softening due to friction heating may be the dominant reason for the increased wear volume loss.

**Figure 8:**
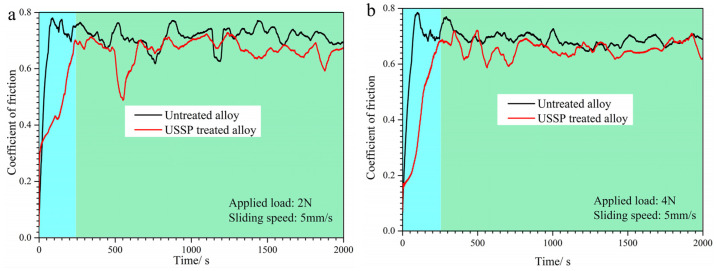

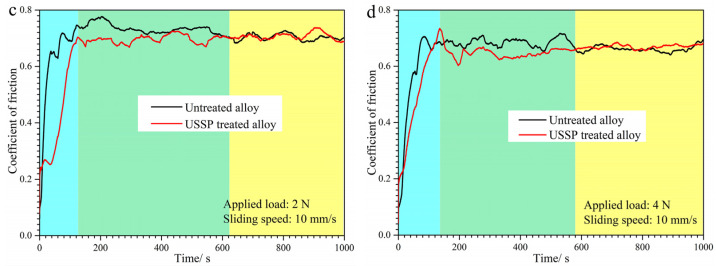
The COF curves of Ti-50.8Ni alloys before and after USSP treatment under different wear conditions: (**a**) 5 mm/s, 2 N; (**b**) 5 mm/s, 4 N; (**c**) 10 mm/s, 2 N; (**d**) 10 mm/s, 4 N.

To better understand the tribological behaviors of Ti-50.8Ni alloy, the in-time COF curves have been recorded as the function of time during the wear process, as shown in Figure 8. The COF curves of Ti-50.8Ni alloy are composed of the run-in stage and steady-friction stage. The run-in stage of the untreated alloy is shorter, which may be due to the smoother surfaces. On the conditions of 5 mm/s, the COF of the USSP-treated alloy is always lower than that of the untreated alloy and the curves tend to be smoother under the higher load of 4 N. However, when the sliding speed is 10 mm/s, the steady-friction stage can be further divided into two parts: the USSP-treated alloy holding the lower COF in the former stage and two alloys holding the similar COF in the latter stage. This indicates that the friction behaviors tend to be similar for the two alloys with the increase in wear time.

### 3.4. Wear Surfaces

**Figure 9:**
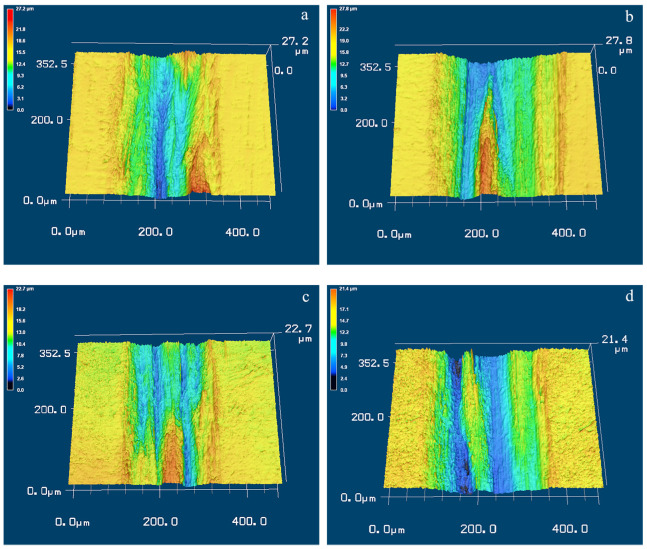
The 3D morphologies of the wear tracks of Ti-50.8Ni alloys on the condition of 5 mm/s: (**a**) Untreated alloy, 2 N; (**b**) Untreated alloy, 4 N; (**c**) USSP-treated alloy, 2 N; (**d**) USSP-treated alloy, 4 N.

To further unravel the wear mechanism of Ti-50.8Ni alloy, the three-dimensional morphologies of the wear tracks on the condition of 5 mm/s have been shown in Figure 9. As shown, apparent wear grooves can be observed on the alloy’s surfaces after dry sliding wear. The width of the groove shows good agreement with the wear volume loss. Intriguingly, a few ridges appear inside the wear grooves, and these ridges seem to become more visible under a higher applied load. During the wear process, severe deformation occurs on the surface of Ti-50.8Ni alloy except for the regular abrasion. The stresses between the GCr15 ball and the sample surface can result in the martensite transformation, which definitely helps to improve the surface hardness of the Ti-50.8Ni alloy. The improved hardness is beneficial to enhancing the hindering effect of micro-cutting behaviors from the wear ball. Consequently, the wear resistance can be enhanced correspondingly, and the wear grooves tend to be uneven. Plastic deformation is still retained underneath the wear tracks after sliding wear. Therefore, the higher applied load is supposed to contribute to more apparent deformed ridges inside the grooves [41].

**Figure 10:**
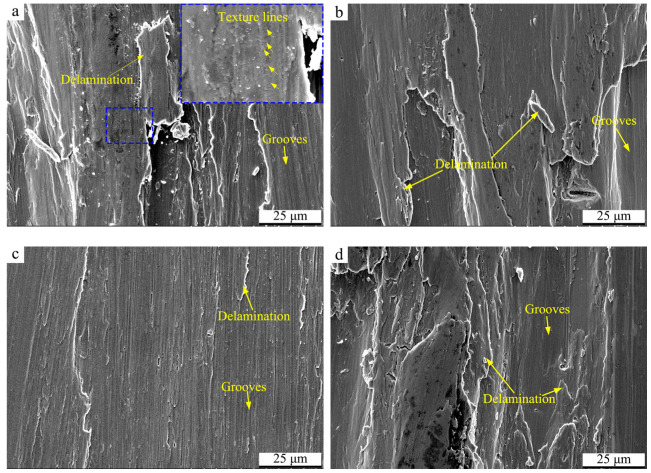
SEM micrographs of the wear tracks of Ti-50.8Ni alloys on the condition of 5 mm/s: (**a**) Untreated alloy, 2 N; (**b**) Untreated alloy, 4 N; (**c**) USSP-treated alloy, 2 N; (**d**) USSP-treated alloy, 4 N.

Figure 10 displays the SEM micrographs of the wear surfaces of Ti-50.8Ni alloys on the condition of 5 mm/s. As shown, grooves dominate the wear damage form, resulting from the micro-cutting of the counterpart ball. This indicates the predominant wear mechanism is abrasive wear [40]. In addition, delamination can also be found on the wear surface due to the reduplicative rolling deformation [42]. By comparison, the wear surface of the USSP-treated Ti-50.8Ni alloy is the smoothest in the case of 2 N, which may be attributed to the higher surface hardness during the wear process. When the applied load is 4 N, obvious tearing morphology can be observed on the wear surfaces of Ti-50.8Ni alloys except for the micro-grooves and delamination. This indicates that adhesive behaviors may take place under the relatively higher hardness of 4 N. On the other hand, a large number of short textures perpendicular to the sliding direction can be observed on the wear surface of the untreated alloy in the case of 2 N, which may be caused by the reduplicative deformation during the sliding wear. This kind of morphology can also be the evidence of the relatively higher COF.

**Figure 11:**
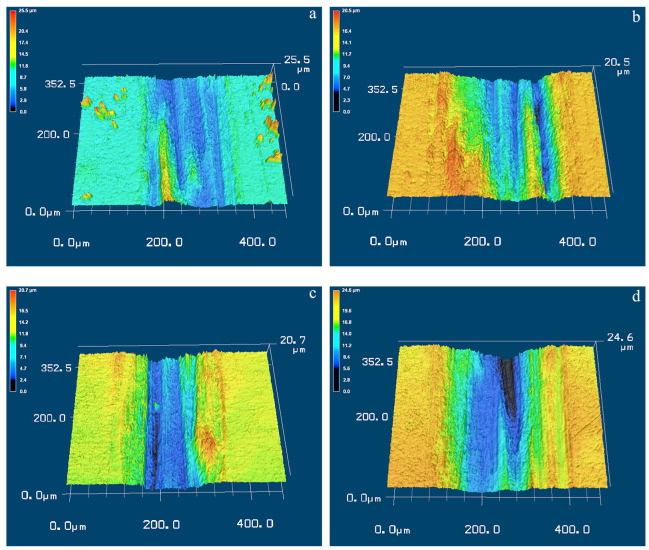
The 3D morphologies of the wear tracks of Ti-50.8Ni alloys on the condition of 10 mm/s: (**a**) Untreated alloy, 2 N; (**b**) Untreated alloy, 4 N; (**c**) USSP-treated alloy, 2 N; (**d**) USSP-treated alloy, 4 N.

On the condition of 10 mm/s, the wear grooves and inside ridges can also be observed on the wear surface of Ti-50.8Ni alloy. From Figure 11a,b, it can be found that the wear grooves seem shallower for the untreated alloys compared the those on the condition of 5 mm/s. This agrees well with the fact that the wear volume loss of untreated alloy can be reduced when the sliding speed increases to 10 mm/s. However, the depth of wear grooves on the USSP-treated alloy is obviously larger. And the bottom of the grooves seems to be smoother, implying the alloy’s resistance to abrasion is weaker. Consequently, the wear volume loss can be increased with the increase of sliding wear speed.

**Figure 12:**
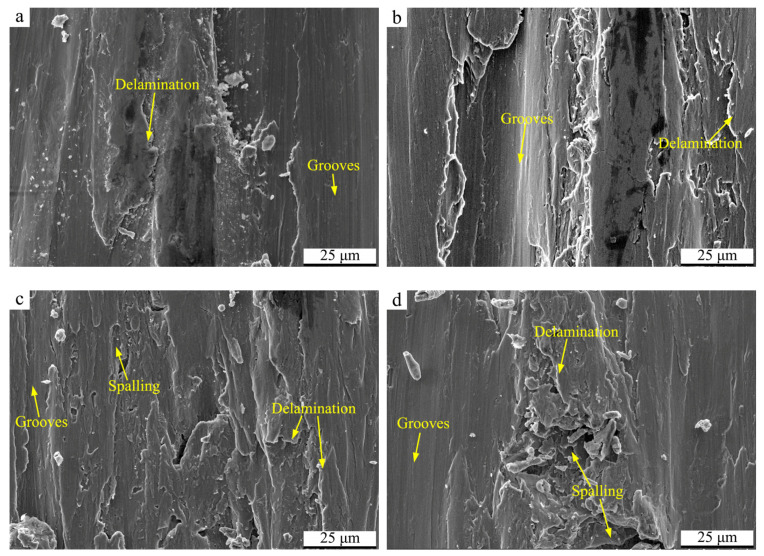
SEM micrographs of the wear tracks of Ti-50.8Ni alloys on the condition of 10 mm/s: (**a**) Untreated alloy, 2 N; (**b**) Untreated alloy, 4 N; (**c**) USSP-treated alloy, 2 N; (**d**) USSP-treated alloy, 4 N.

To clarify the variation of tribological behaviors, Figure 12 displays the SEM micrographs of the wear surface on the condition of 10 mm/s. By comparison, the overall wear damage forms are similar to those in the case of 5 mm/s with micro-cutting and delamination dominating the wear process. However, the delamination morphology is more apparent on the wear surfaces. In addition, the spalling can be observed in local regions on the wear surfaces of the USSP-treated alloy, which may be the crucial reason for the higher wear volume loss. Both the martensite transformation and softening originating from friction heating operate during the wear process of Ti-50.8Ni alloy in case of higher sliding speed [11]. As for the USSP-treated alloy, the latter plays the predominant role so that the wear resistance decreases correspondingly. This is supposed to be attributed to the alloy’s potential to absorb the deformation energy decreases after the USSP treatment. However, the martensite transformation can be facilitated in case of higher sliding speed, contributing to the increased surface hardness. On the other hand, the wear grooves in the case of 4 N are obviously deeper than those in the case of 2 N, as shown in Figure 12a,b, and the few micro-cracks can be observed to appear on the tribo-layer of Ti-50.8Ni alloy. Consequently, the wear volume loss is relatively higher under a higher applied load. As above, the USSP-treated Ti-50.8Ni alloy shows poorer tribological performance on the condition of a higher sliding speed of 10 mm/s.

In conclusion, it can be established that the USSP treatment can have dual effects on the tribological performance of Ti-50.8Ni SMA alloy. When the sliding speed is 5 mm/s, the USSP treatment can obviously improve the wear resistance as well as decrease the COF. This is mainly attributed to the higher hardness and facilitation of the USSP treatment on the stress-induced martensite transformation. However, when the sliding wear speed increases to 10 mm/s, the wear resistance of the USSP-treated sample would reversely decrease owing to the severe delamination and spalling.

## 4. Conclusions

This work investigates the effects of ultrasonic surface shot peening on the microstructure, hardness, and tribological behaviors of Ti-50.8Ni SMA alloy. The results indicate that the USSP treatment is an effective method to modify the tribological properties of Ti-50.8Ni SMA, especially under relatively lower sliding speeds. Compared to the other improved methods, the USSP treatment is more economical and simple, considering the engineering applications. The following main conclusions can be drawn:

(1) After the USSP treatment, the phase composition has little change, with the B19′ phase slightly increasing; additionally, the main B2 phase grain can be refined to some degree;
(2) The USSP treatment can dramatically improve the surface hardness, with the highest hardness of 541 HV obtained under the test load of 25 g; however, the hardness would decline quickly with the further increase of the test load.
(3) In the case of 5 mm/s, the wear volume loss and COF are much lower for the USSP-treated alloy, implying the improvement of tribological performance; however, when the sliding wear speed increases to 10 mm/s, the wear resistance of the USSP-treated alloy reversely decreases while that of the untreated alloy increases. Stress-induced martensite transformation and degeneration resulting from frictional heating dominate the dry sliding wear mechanism of Ti-50.8Ni alloy.

## Figures and Tables

**Table 1:** The specific parameters of sliding wear testing.

Parameters	Sliding Speed/mm/s	Applied Load/N
Group 1	5	2
Group 2	5	4
Group 3	10	2
Group 4	10	4

**Table 2:** The phase proportion of Ti-50.8Ni alloy before and after USSP treatment (%).

Phase	Untreated Alloy	USSP-Treated Alloy
TiNi (B2)	79.63	71.25
TiNi (B19′)	0.69	3.75
Ni4Ti3	3.14	2.82
Ni3Ti	0.03	0.04
NiTi2	0.24	0.22
Unresolved areas	16.27	21.92

## Data Availability

Data are contained within the article.
